# Novel Coronavirus and Common Pneumonia Detection from CT Scans Using Deep Learning-Based Extracted Features

**DOI:** 10.3390/v14081667

**Published:** 2022-07-28

**Authors:** Ghazanfar Latif, Hamdy Morsy, Asmaa Hassan, Jaafar Alghazo

**Affiliations:** 1 Computer Science Department, Prince Mohammad Bin Fahd University, Khobar 34754, Saudi Arabia; 2 Department of Computer Sciences and Mathematics, Université du Québec à Chicoutimi, 555 Boulevard de l’Université, Chicoutimi, QC G7H 2B1, Canada; 3 Department of Applied Natural Sciences, College of Community, Qassim University, Buraydah 52571, Saudi Arabia; h.morsy@qu.edu.sa; 4 Department of Electronics and communications, College of Engineering, Helwan University, Cairo 11792, Egypt; 5 Faculty of Medicine, Helwan University, Helwan 11795, Egypt; asmaa49eg@gmail.com; 6 Department of Electrical and Computer Engineering, Virginia Military Institute, Lexington, VA 24450, USA; alghazojm@vmi.edu

**Keywords:** chest CT scan, COVID-19 detection, deep learning features, convolutional neural network (CNN), common pneumonia, novel coronavirus pneumonia

## Abstract

COVID-19 which was announced as a pandemic on 11 March 2020, is still infecting millions to date as the vaccines that have been developed do not prevent the disease but rather reduce the severity of the symptoms. Until a vaccine is developed that can prevent COVID-19 infection, the testing of individuals will be a continuous process. Medical personnel monitor and treat all health conditions; hence, the time-consuming process to monitor and test all individuals for COVID-19 becomes an impossible task, especially as COVID-19 shares similar symptoms with the common cold and pneumonia. Some off-the-counter tests have been developed and sold, but they are unreliable and add an additional burden because false-positive cases have to visit hospitals and perform specialized diagnostic tests to confirm the diagnosis. Therefore, the need for systems that can automatically detect and diagnose COVID-19 automatically without human intervention is still an urgent priority and will remain so because the same technology can be used for future pandemics and other health conditions. In this paper, we propose a modified machine learning (ML) process that integrates deep learning (DL) algorithms for feature extraction and well-known classifiers that can accurately detect and diagnose COVID-19 from chest CT scans. Publicly available datasets were made available by the China Consortium for Chest CT Image Investigation (CC-CCII). The highest average accuracy obtained was 99.9% using the modified ML process when 2000 features were extracted using GoogleNet and ResNet18 and using the support vector machine (SVM) classifier. The results obtained using the modified ML process were higher when compared to similar methods reported in the extant literature using the same datasets or different datasets of similar size; thus, this study is considered of added value to the current body of knowledge. Further research in this field is required to develop methods that can be applied in hospitals and can better equip mankind to be prepared for any future pandemics.

## 1. Introduction

The World Health Organization (WHO) announced the Severe Acute Respiratory Syndrome Coronavirus 2 (SARS-CoV-2) as a pandemic on 11 March 2020. The first reported case was in December 2019; due to the contagious nature of the virus, it started to spread all over the world. SARS-CoV-2, which gives rise to Coronavirus Disease 19 (COVID-19), is a type of coronavirus that causes diseases in mammals and birds. COVID-19 belongs to a family of viruses that can cause pneumonia in humans with symptoms similar to cold, fever, and respiratory issues [1,2].

COVID-19 was discovered as a new virus at the end of 2019. Some studies have confirmed that the virus was transmitted through bats and cats to humans [3]. The spread of COVID-19 can be minimized by isolating infected people from noninfected people. There are some signs of infection, such as respiratory symptoms and cough, which can end with fever and dyspnea. In some cases, the virus infection causes pneumonia and severe acute respiratory syndrome, potentially leading to death [4]. The treatment of COVID-19 disease starts with prescribing medication for patients and getting home rest; however, in severe cases, patients need to be hospitalized, occasionally needing admission to an intensive care unit (ICU). Some patients are cured after hospitalization, while others unfortunately suffer and lose their lives. As of 16 March 2022, there were 460,280,168 confirmed cases of COVID-19, including 6,050,018 deaths, as reported by the WHO [5]. According to the latest studies, 15% of all infected people need to be hospitalized, 5% of which need admission to an intensive care unit (ICU). The number of ICU beds per person is a significant indicator of the health care system of a certain country.

Due to the quick spread of the COVID-19 pandemic spread, the average number of beds per 1000 people is less than the required number for those in urgent need of hospitalization. There are two types of beds, one for moderate cases and recovery and the other for ICU cases. Most countries cannot meet the minimum requirements for hospitalization, especially developing countries, with even advanced countries also suffering from a shortage of ICU beds and ventilators during peak phases, which are needed in severe cases. The health systems all over the world were not prepared for this pandemic. As a result, some patients could not be hospitalized and some of them died on the street. The current practice for diagnosing COVID-19 includes a sample from the person’s nose or throat, which is then subjected to a polymerase chain reaction (PCR) test. This test is used for contact tracing and controlling the spread of COVID-19 by isolating infected patients from healthy individuals. This test is applicable to both asymptomatic and symptomatic individuals, e.g., high body temperature (above 38 °C) and coughing [6]. Computed tomography (CT) and X-ray scans are two techniques used for imaging the human chest to visually diagnose COVID-19 pneumonia patients and discriminate them from non-COVID-19 pneumonia patients and healthy cases. Fortunately, the imaging process can be stored and processed using computer program tools [7], which eases the pressure on healthcare professionals.

An early diagnosis of COVID-19 results in the development of an appropriate treatment plan and containment of the spread of the virus, which can help both the patient and the community. COVID-19 causes infection in the lungs and upper respiratory tract. Despite the development of off-the-counter diagnosis tests, which are not reliable and still require hospital testing for an exact diagnosis, the problems of the number of patients being tested and the lack of medical personnel required to perform the tests and then accurately diagnose the results have not been solved. In some countries, the lack of medical personnel is taking a toll on the health system, which has increased with the COVID-19 pandemic. The exponential rise in COVID-19 patients is overwhelming the global healthcare system, which is more apparent in developing countries. Due to the resemblance of the symptoms of COVID-19 to the common cold, it is nearly impossible to test every patient that exhibits such symptoms. If a system is available that could potentially diagnose COVID-19 and other respiratory diseases automatically without human intervention, this could ease the pressure on healthcare systems and personnel. This is possible with the use of deep learning and machine learning methods. Chest X-rays or CT scans of the lungs can be made readily available, whereby machines will be able to accurately diagnose not only COVID-19 but also other upper respiratory diseases such as bacterial and viral pneumonia, representing the motivation of this study. Although much research has been conducted on this subject, an ultimately accurate solution has still not been attained for many reasons, such as the lack of a standardized dataset.

In this paper, the aim was to develop a system utilizing optimized deep learning or machine learning techniques for detection of the novel coronavirus and common pneumonia without the intervention of humans. The ultimate goal was to develop a method that can diagnose these diseases with higher accuracies than those reported in the extant literature using similar methods and/or similar datasets.

## 2. Recent Studies

Since the beginning of COVID-19, scientists all over the world have been working on four aspects of this infectious virus: diagnosis, vaccination, treatment, and intervention against disease spread. While scientists in the medical field have been tackling these issues from a purely chemical and medical aspect, computer scientists have been working on diagnosis using machine learning techniques. Many research articles have also been written on the subject of the analysis of the spread of the disease and predictions of the spread of the disease using machine learning methods. The first requirement for using machine learning in COVID-19 diagnosis is the development of datasets. In [8], the authors developed a dataset from various websites and publications to obtain 123 frontal view X-rays. In [9], the authors developed a homogeneous dataset they named COVIDGR-1.0, containing a balanced number of X-rays for all levels of severity of COVID-19 cases. COVIDGR-1.0 is composed of 426 negative cases and 426 positive cases for a total of 852 images. Other datasets used in these studies are available in [10,11,12,13]. Other research involving machine learning tackled other aspects of COVID-19 such as a tweet dataset [14] and face mask detection [15].

In this paper, we primarily focus on the diagnosis of COVID-19 using X-ray images; thus, the remainder of the literature review highlights research conducted on the diagnosis of COVID-19. In [16], the authors proposed the use of transfer learning with deep learning methods to detect COVID-19 using X-ray, ultrasound, and computerized tomography (CT) scans. They applied a VGG-19 model with specific parameters. They further proposed a preprocessing phase to minimize unwanted noise in the images. They concluded that ultrasound images were better for detection than CT scans and X-rays. They reported a precision of 100% for ultrasound, 84% for CT scans, and 86% for X-rays. They used the National Institute of Health chest X-ray dataset, the dataset specified in [17], and the COVID-CT dataset. In [18], the authors proposed several approaches for the detection of COVID-19 using chest X-rays. These included the fine-tuning of pretrained convolutional neural networks (CNNs), deep feature extraction, and end-to-end training of a developed model. They used ResNet 18, VGG19, VGG16, ResNet101, and ResNet50 for deep feature extraction and fine-tuning. For the classification phase, they used support vector machines (SVMs) with various kernel functions to include quadratic, linear, Gaussian, and cubic functions. They also proposed a CNN method and used a dataset of 200 healthy images and 180 COVID images. They reported an average accuracy of 94.7%. In [19], the authors proposed deep learning methods for the detection of COVID-19 using chest X-ray images. They proposed the use of a deep CNN network for the detection of COVID-19 on a dataset consisting of 1000 images that they produced relying on an original dataset and augmentation. They reported an F-measure range between 95% and 99%. They reported accuracy of 88.43% and 94.8% for Jordan and Australia, respectively. In [20], the authors proposed the use of X-rays for the initial diagnosis of COVID-19 patients selected for further reverse transcription polymerase chain reaction (RT-PCR) testing. They proposed a system called CovidAID using deep neural networks using a publicly available dataset [8]. They used CheXNet, which consists of 121 layers of dense convolutional networks (DenseNet). They tested the system for three classes and four classes, reporting accuracies of 90.5% and 87.2%, respectively. In [21], the authors proposed a multitask deep learning algorithm used on both X-rays and CT scans for the diagnosis of COVID-19. They used the dataset in [7] combined with the dataset in [8]. They propose the use of an inception residual recurrent CNN with transfer learning (TL) for the detection of COVID-19 and then the use of an NABLA-N model for segmenting the infected region. They reported a testing accuracy of 98.78% on CT scans and 84.67% on X-ray images. In [22], the authors proposed the use of CNN for COVID-19 detection and achieved an accuracy of 85%. This was achieved using the dataset collected by the authors from three publicly available datasets: (1) IEEE COVID Chest X-ray Dataset (2) COVID-19 Chest X-ray Dataset Initiative, and (3) COVID-19 Radiography Database. However, the authors argued that synthetic datasets can add to the accuracy of CNN models; thus, they proposed the use of an auxiliary classifier generative adversarial network (ACGAN) to generate synthetic chest X-rays. Using the dataset with the added synthetic images, they reported that the CNN accuracy increased to 95%. In [23], the authors proposed a novel CNN model consisting of 22 layers, called CoroDet, for the automatic diagnosis of COVID-19 using chest X-rays and CT scans. Their proposed system was designed for binary classification and multiclass classification, i.e., three-class (pneumonia, normal, and COVID-19) and four-class (bacterial pneumonia, viral pneumonia, normal, and COVID-19) classification. They reported classification accuracies of 99.1%, 91.2%, and 94.2% for binary, four-class, and three-class classification, respectively. They also compiled their dataset from publicly available datasets, which they claimed to be the largest dataset as of writing the paper. In [24], the authors took the poster anterior (PA) view of chest X-ray images of COVID-19 infected patients and healthy patients. They compared the performance of three deep learning methods in the detection of COVID-19, namely, Inception V3, Xception, and ResNeXt. They collected a dataset consisting of 6432 images from Kaggle and used 5467 images for training and 965 for validation. They obtained the highest accuracy using Xception with an accuracy of 97.97%. In [25], the authors proposed the use of advanced deep learning models along with transfer learning strategies utilizing a custom input size for each deep learning model to achieve the best performance. They used CT image datasets, namely, COVID19-CT and SARS-CoV-2 CT-Scan, reporting average accuracies of 92.9% and 99.4%, respectively. They further used visualization techniques to segment and get an accurate localization of the COVID-19-infected regions. In [26], the authors proposed a machine vision method for the detection of COVID-19 using chest X-rays. They proposed that the features be extracted using CNN and histogram-oriented gradient (HOG). They employed the use of a modified anisotropic diffusion filter method (MADF) to preserve edges and reduce noise. They also used watershed segmentation to segment the region of the image that showed COVID-19 infection. The classification was achieved through a CNN using VGGNet. They used three publicly available datasets in their study. They reported an accuracy of 99.49% for binary classification (infected with COVID-19 or healthy). In [27], the authors proposed an optimized deep learning method for the automatic diagnosis and classification of COVID-19 using X-ray images. They used transfer learning algorithms such as DenseNet, VGG16, VGG19, GoogleNet, and AlexNet. They reported the highest accuracy of 95.63% using VGG16.

Many reviews have also been written regarding machine learning algorithms and their use in the detection of COVID-19. In [28], the authors conducted a survey of the published deep learning methods used for the detection of COVID-19 using lung images. Their survey included the datasets used, methods, and evaluation metrics. They also compared the published metrics and results. They concluded that the best strategies were either transfer learning or fine-tuning. In [29], the authors reviewed the published methods for COVID-19 detection.

Machine learning (ML) algorithms are not only used for COVID-19 detection. Research is ongoing on the use of ML algorithms for the diagnosis and treatment of many other medical conditions. For example, in [30] the authors used deep learning for brain tumor diagnosis and detection, and, in [31], the authors used deep learning methods for diabetic retinopathy detection from fundus images. In addition, segmentation of images related to medical images is an important aspect related to the use of machine learning in the medical field [32,33].

## 3. Deep Convolutional Neural Networks

Convolutional neural networks (CNNs) and deep learning (DL) are techniques in artificial intelligence (AI) used to process image datasets to detect objects called classes in the dataset. These computerized techniques have very high accuracy in identifying objects and are also very fast compared to humans. Medical imaging such as computed tomography (CT) chest scans can be grouped and divided into datasets for classes such as COVID-19 and non-COVID-19 pneumonia and healthy cases [34,35]. These classes may include hundreds or even thousands of images for each class, which are then used for training and testing purposes. The main reasons for utilizing ML and DL are the need for fast and accurate results in identifying COVID-19-infected people from healthy people and the shortage of healthcare professionals to diagnose and examine each person for coronavirus [36]. It should be mentioned here that CNN is a type of deep learning which is mainly applied to images but can also been used for other tasks such as speech recognition and natural language processing. CNNs that are beyond a certain depth of layers can be referred to as deep learning (DL) networks. There are other neural network-based architectures such as recurrent neural networks and autoencoders which are also considered under deep learning. CNNs and DL need a large dataset from different patients and healthy people to be processed for feature extraction using CNN networks.

The CNN network, in its simplest form, consists of an input layer, output layer, and hidden layer. The input layer consists of several neurons, with each neuron representing a one-pixel value from the input image. In CNN, each group of neurons is connected to a single neuron in the next hidden layer. This is different in fully connected layers, where each neuron is connected to all neurons of the next hidden layer. The input neurons have weights to reflect the effect of those neurons, and a bias is added to the sum to modify the final sum. This process (forward propagation) is repeated for all hidden layers until reaching the output layer. During the training process, the results are fed back (backpropagation) to maximize the accuracy of identifying objects and minimize the output cost, which in this case is the error. An activation function is used for the sum of the neurons in each next layer neuron. This activation function should be differentiable so that the output error can be minimized. Examples of activation functions are linear, sigmoid, Tanh, ReLU, and softmax [37]. The method of activation function and differentiation is stochastic gradient descent, which is used to minimize the output cost using backpropagation. The weight of each neuron is updated during the training with a value called the learning rate which ranges between 0.0 and 1.0. The process of training is repeated for a number of epochs and a batch size determined at the beginning of the training process. The learning rate, number of epochs, and batch size are hyperparameters determined before starting the training process. Hyperparameter tuning is applied in order to optimize the model.

## 4. Experimental Dataset

The dataset used in this work was a combination of several publicly available datasets named CC-CCII [38]. The original dataset was a collection of open-access CT scan datasets. The dataset had a total of 85,611 chest CT images of 2711 patients containing three classes: normal, novel coronavirus pneumonia (NCP), and common pneumonia (CP). All images had a resolution of 512×512 pixels. Approximately 41% of the dataset images belonged to the NCP class, compared to around 26% for CP images and nearly 33% for normal images. Table 1 shows a summary of the experimental dataset along with sample images. The total number of images was 35,191 from 932 patients for CP, 21,872 from 929 patients for NCP, and 28,548 from 850 patients for normal.

## 5. Method

**Figure 1:**
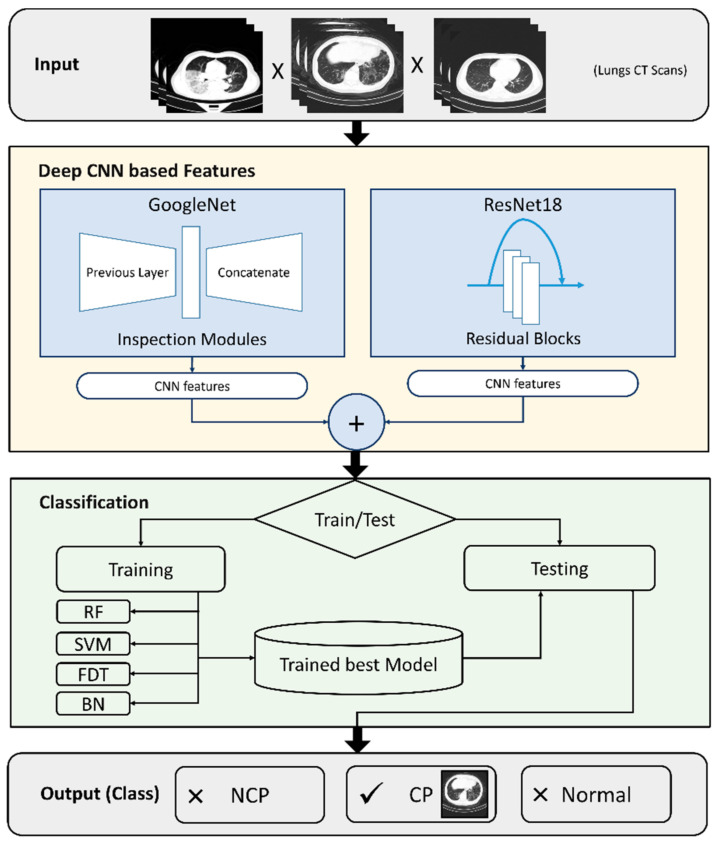
Workflow of the proposed system.

In this article, we propose the use of deep learning algorithms for feature extraction. Figure 1 details the proposed enhanced process for automatic COVID-19 detection and diagnosis. The dataset is input to both GoogleNet and ResNet18 for feature extraction. In the proposed method, GoogleNet extracts a total of 1000 features, and ResNet18 extracts a total of 1000 features. Hybrid features from the selected models are extracted using the stochastic gradient descent with momentum (SGDM) optimizer with a learning rate of 0.0001 and batch size of 200 over 100 epochs. The combined 2000 features are then input to the second phase of training and testing, where the data are split into 80% training and 20% testing. The 20% testing dataset is further split into 80% testing and 20% validation. During this phase, training is performed using four well-known classifiers, namely, random forest (RF), support vector machine (SVM), fast decision tree (FDT), and Bayesian network (BN). The portion of the dataset dedicated to testing is then applied to the classifiers to be classified as NCP, CP, or normal. The other portion is used to validate the results and process. Among the contributions of this work is the proposal of extracting features from various deep learning techniques, with each technique extracting unique features that, when combined, increase the performance of the detection and diagnosis to a level that can practically be used within hospitals to assist medical professionals in the accurate diagnosis of COVID-19 using chest X-rays. The details of the phases are explained below.

### 5.1. Deep Learning Features

Artificial neural networks (ANNs) form the fundamental principles of deep learning (DL) methods [39]. The inspiration for the structure of these networks originated from the inner workings of neurons in the human brain. The most important element in an ANN is the perceptron, whose output is mathematically formulated as shown in Equations (1)–(3).

(1) $$y=f\left(w_{0}+\sum X^{T}\times W\right)$$

(2) $$y=f\left(z\right)$$

(3) $$where\;z=w_{0}+\sum X^{T}\times W$$

where *W* is the weight matric, *X* is the input, *w*_0_ is the bias, and *f* is the nonlinear function. Basically, on the basis of the input, the ANN is trained to predict the output using the various neurons within the system. The main requirement is the quantity of the input size that allows for the system to capture features of the input to allow accurate prediction. The ANN can have multiple layers, with each consisting of perceptrons, which are referred to as hidden layers.

In deep neural networks, there are multiple hidden layers, and the input dataset must be really large for the network to be able to learn the features of the input data. For image processing, a certain type of deep learning network is used called the convolutional neural network (CNN) [40]. In CNNs, filters within a convolutional operator are used to create a feature map from the input images, where each filter has a specific function of capturing an image feature. This does not exist within a fully connected neural network. A rectified linear unit (ReLU) exists within the activation layer to make the system nonlinear [41]. Convolutional layers replace the hidden layers in an ANN. These layers are able to capture high-, medium-, and low-level features of the input images. In order to reduce the dimensions of the input image, pooling is performed. Lastly, the image of the reduced feature set is flattened and inputted to the fully connected layer for prediction.

#### 5.1.1. GoogleNet

**Figure 2:**
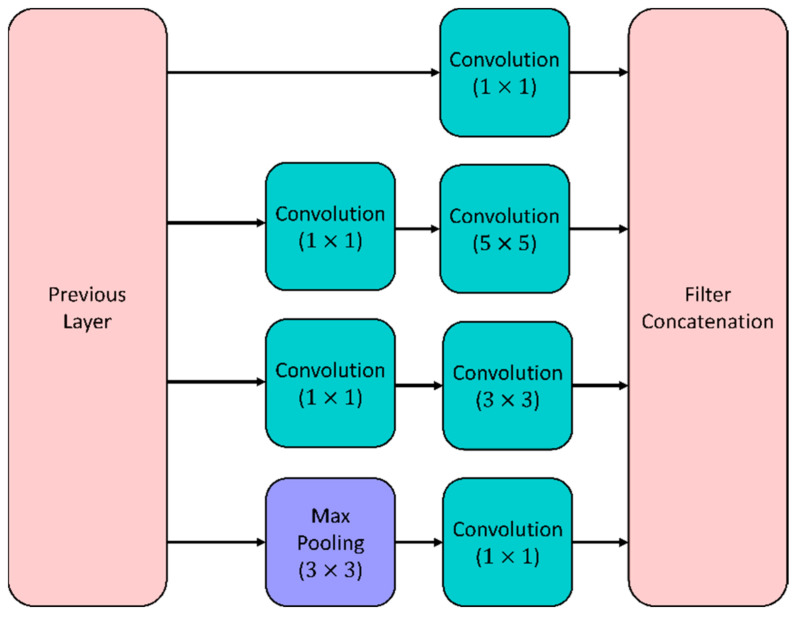
Inception module for the GoogleNet model.

A well-known CNN is GoogleNet, consisting of 22 deep layers with computational resources utilizing inception modules [42]. Enhancement of the depth and width of the network is performed using the inception modules, which also assist in capturing the features at all scales. Various spatial and local features are captured by the inception module, which consists of various-sized convolutional layers. Figure 2 shows the inception module. The dimensions of the input are reduced using a 1×1 convolutional layer, resulting in the extraction of local cross-channel features. Spatial features are captured using 3×3 and 5×5 convolutional layers. The dimensions of the input are also reduced using the pooling layer that exists in the inception module. Figure 2 provides an overview of GoogleNet, which utilizes the inception module.

#### 5.1.2. ResNet18

Another type of CNN is the residual neural network (ResNet), which gets rid of some layers in the network through a process that skips connections [43]. This process assists in solving the vanishing gradient problem in CNN and results in reduced training time. Between the skipped connections are nonlinear activation functions with batch normalization existing between the shortcut connections. The weights of the jump connections are calculated using a weight matrix. Expansion is applicable in further stages of the network after learning the features of the inputs.

**Figure 3:**
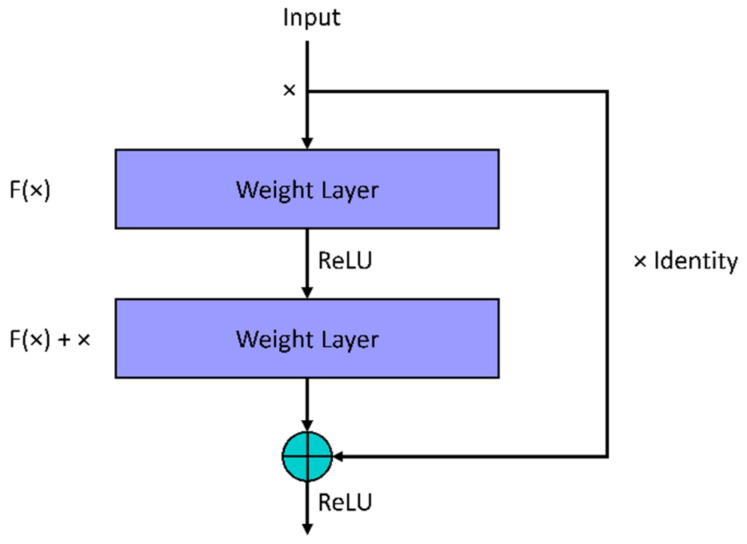
Building block of a residual network.

The basic building blocks of ResNet are shown in Figure 3. Throughout the network, multiple instances of the residual blocks are integrated. The map learning from *x → f(x)* is performed in the convolutional neural network. The formalization of the mapping in the residual network block is achieved through a feedforward neural network containing shortcut connections referred to as skip or jump connections, i.e., *x → f(x) + g(x)*, where *g(x)* is the identity connection with the condition that the output and input dimensions match and zero-padding if they do not. Equation (4) shows the resulting residual block for the stacked layers in the ResNet with matching dimensions.

(4) $$y=f\left(x,\left\{W_{i}\right\}\right)+x$$

where $f\left(x,\left\{W_{i}\right\}\right)$ represents the mapping of the convolution layers learned during the training process. In [43], the authors used ResNet, consisting of 3×3 filters with a stride of 1, a 1×1 filter in the pooling layer, one fully connected layer at the final stage, and a final softmax layer solely for classification. In this work, we propose the use of 17 convolutional layers in ResNet18 to extract 1000 features.

### 5.2. Classification

Unstructured data are classified into groups according to their different features through classification. Classification techniques are utilized in object recognition. These data are defined as datasets which have many forms of data, such as digits, letters, animals, or even groups of X-ray images. In this sense, classification refers to the process of categorizing datasets according to the format of data to be processed. The required information from the datasets is extracted first before classification takes place. Hence, the datasets are analyzed and processed to achieve the required classification. Two main classification techniques exist: supervised and unsupervised. The supervised classification technique is based on labeled data and is known as predictive or directed classification. In this technique, a training dataset with classified data with labels is fed to the system for training and analyses to achieve maximum accuracy. The features of these data are learned, and the system is ready for new unlabeled data to be processed. The unsupervised classification technique is based on unknown information of the datasets and is known as descriptive or undirected classification. In this technique, the unlabeled data are fed to the system and, after processing and analysis, labels can be assigned to different classes [44]. In this section, we introduce some of the supervised classification techniques.

#### 5.2.1. Fast Decision Tree (FDT)

**Figure 4:**
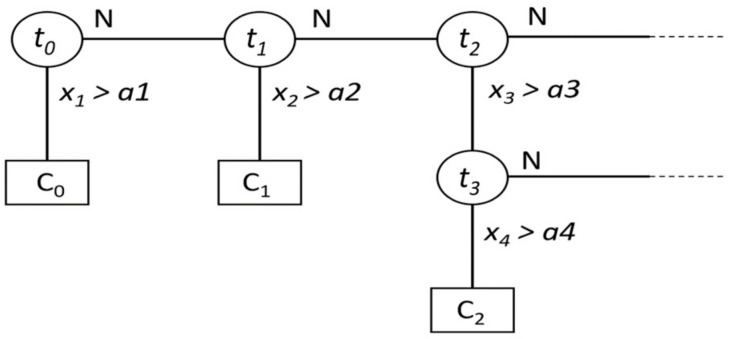
Fast decision tree nodes and their decision outcomes.

Among the most effective learning algorithms is the fast decision tree (FDT) method, because of its many attractive features, simplicity, and ability to process different data types. This algorithm is based on a set of labeled datasets containing attribute values and class labels. It is a greedy algorithm that starts from top-down with an empty tree and a recursive process. The time complexity of a decision tree algorithm such as C4.5 is O (m·n^2^), where m is the number of training datasets, and n is the number of attributes. This algorithm outperforms the Bayesian algorithm on larger datasets, while the Bayesian algorithm behaves better on smaller datasets. There are two approaches to the fast decision tree algorithm, namely, a restricted model space search and a powerful search heuristic [45]. Figure 4 shows part of an FDT with a pattern $x_{i}=\left(x_{i1},{\;x}_{i2},\;\ldots ,{\;x}_{\mathrm{in}}\right)$, n-dimensional space, and node t_i_. For each threshold t_i_, a decision is made for a pattern x_i_ to identify its class. The process continues until the training dataset is exhausted. For example, if x_1_ > a1, where a1 is a threshold, then x_1_ belongs to class C_0_, and the process keeps going.

#### 5.2.2. Random Forest (RF)

**Figure 5:**
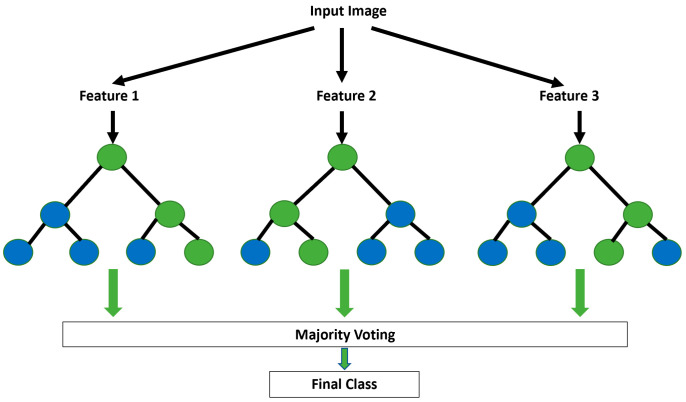
Random forest algorithm input and output process based on majority voting.

A random forest is a decision tree ensemble where multiple decision trees are combined to make the strongest model possible. In terms of robustness, accuracy, and overfitting, this derived model is better than its constituent models. Random forests employ ensembles of decision trees with “bagging methods” to obtain classification and regression outputs [46]. In classification, the output is processed using majority voting (see Figure 5), whereas, in regression, the system calculates the mean. When some data are missing, the algorithm can automatically handle them and maintain accuracy even when large proportions of the data are missing. The random forest technique can be used for nonparametric data. In this technique, no extensive training or design is required. The only disadvantage of this technique is its complexity due to the number of trees created and combined at the output, which also leads to a time-consuming training process.

#### 5.2.3. Support Vector Machine (SVM)

**Figure 6:**
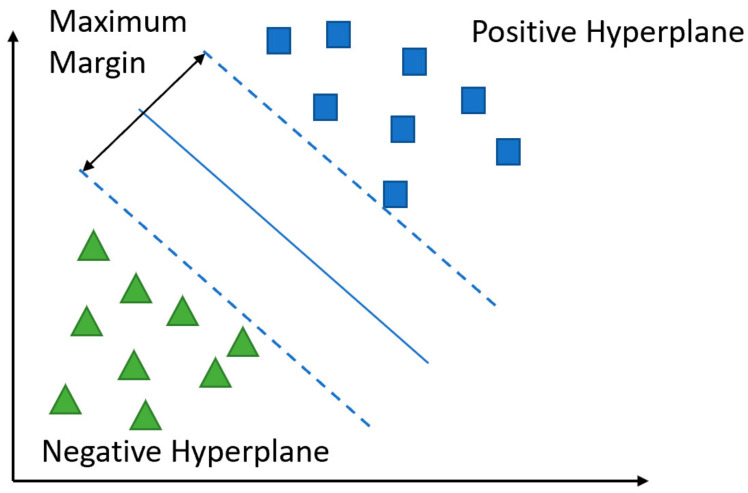
SVM positive and negative hyperplanes with the maximum margin.

Support vector machines are used to classify groups of datasets into different classes in hyperplanes. The SVM is among the most popular and powerful learning algorithms. With SVMs, the main objective of the optimization is the maximization of margins. The margin is equal to the distance between the separating hyperplane or decision boundary [47]. The output of the *i* (1, …, *N*) dataset can be calculated for the positive and negative hyperplanes using Equations (5) and (6). We can obtain a compact form of these output equations as shown in Equation (7). With some simplification, we can reach the final form in Equation (8).

(5) $$w_{0}+w^{T}x^{\left(i\right)}\geq 1\;if\;y^{\left(i\right)}=1$$

(6) $$w_{0}+w^{T}x^{\left(i\right)}\leq -1\;if\;y^{\left(i\right)}=-1$$

(7) $$y^{\left(i\right)}\left(w_{0}+w^{T}x^{\left(i\right)}\right)\;\geq 1\;\forall_{i}$$

(8) $$\frac{w^{T}\left(x_{diff}\right)}{\left\|w\right\|}=\frac{2}{\left\|w\right\|}$$

where *x_diff_* represents the distance between the positive and negative hyperplane, which is called the margin. The maximation of this margin can be reached by maximizing the value $\frac{2}{\left\|w\right\|}$. Figure 6 shows the positive and negative hyperplanes with the maximum margin.

#### 5.2.4. Bayesian Network (BN)

**Figure 7:**
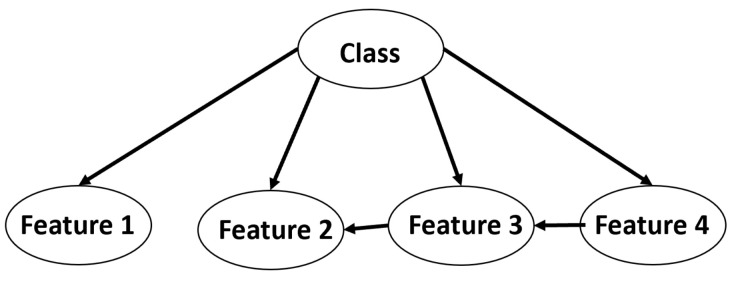
Bayesian network for input class.

BN is a type of probabilistic graphical model (GM) that combines principles from probability theory and graph theory. BNs are intuitively understandable and mathematically rigorous. The joint probability distribution (JPD) can be effectively represented and computed using Bayesian networks [48]. The Bayes theorem (P(A/B) = P(B/A) P(A)/P(B)) can be applied for continuous and discrete cases. Figure 7 shows a Bayesian network for one class having four features, with some of them conditionally depending on others. The relationship between features of the same class follows the Bayes theorem.

## 6. Results

Using the CC-CCII dataset of chest CT scans, the experiments were performed. As seen in the dataset, the numbers of images for normal and NCP were fairly similar. However, the number of images for CP was significantly higher. Nevertheless, we expect that the results obtained can be both significant and practical and not a proof of concept. It is the aim that the proposed enhanced process in this paper and in other similar research can be practically applied in hospitals to assist doctors in the diagnosis process, as doctors would only need to verify the results rather than go through the diagnostic process. As such, to prove the practicality of this process, we chose the following metrics to measure the enhanced process performance; recall, F1-measure, accuracy, precision, confusion matrices, and training time.

In order to prove the significant improvement according to these metrics using the enhanced model proposed in this paper, we performed several experiments, including experiments not using the proposed method, to establish a comparison.

In Table 2, the dataset was applied directly to deep learning algorithms, which we know extract features automatically and perform the classification. We chose the most well-known deep learning algorithms, namely, AlexNet, VGG16, and GoogleNet. We can observe that the accuracy ranged from 97.32% to 98.71%. The highest accuracy, in this case, occurred when using GoogleNet, with a precision of 0.984, recall of 0.972, and F1-measure of 0.978. However, it should be noted that the training time ranged from 25,568 to 54,866 s. A higher accuracy necessitated a longer time for training.

In Table 3, we performed the experiment by extracting the features using GoogleNet where the 1000 features extracted were input to the four classifiers RF, SVM, FDT, and BN according to the percentages mentioned above. The metrics were recorded on the basis of this experiment, and the accuracy ranged from 81.49% to 99.61%. We can note an improvement in the accuracy as compared to the use of deep learning algorithms alone. The accuracy of 99.61% was 0.9% higher than the highest achieved accuracy using GoogleNet alone for both feature extraction and classification. Although this increase is quite significant in the field of machine learning, we can observe that the other metrics also showed improvements. Using SVM, we achieved an accuracy of 99.61%, precision of 0.996, recall of 0.994, F1-measure of 0.997, and training time of 6072 s. We can also note here that the training time significantly improved, ranging from 162 to 6027 s, with the training time increasing with accuracy. However, when comparing the training time observed in Table 1 and Table 2 for the highest achieved accuracy, we can see that the training time was significantly reduced from 54,866 to 6027 s.

In Table 4, we performed an experiment by extracting the features using ResNet18 where the 1000 features extracted were input to the four classifiers RF, SVM, FDT, and BN according to the percentages mentioned above. The metrics were recorded on the basis of this experiment, and the accuracy ranged from 80.14% to 99.86%. We can also note an improvement in accuracy as compared to the use of deep learning algorithms alone (Table 1). The accuracy of 99.86% was 1.15% higher than the highest achieved accuracy using GoogleNet alone for both feature extraction and classification. Again, this increase is quite significant in the field of machine learning. Using SVM as a classifier, the achieved accuracy was 99.86%, with a precision of 0.999, recall of 0.998, F1-measure of 0.999, and training time of 7513 s. All metrics were significantly increased as compared to Table 1, with a particularly significant improvement in training time. The training time now ranged from 133 to 7513 s, with the training time increasing with accuracy. When comparing the training time observed in Table 1 and Table 2 for the highest achieved accuracy, we can see that the training time it was significantly reduced from 54,866 to 7513 s.

In Table 5, the experiment was repeated when the features were extracted using both GoogleNet and ResNet18. The combined features were input to the classifiers RF, SVM, FDT, and BN with the percentages mentioned above. In these cases, we can see that the highest accuracy of 99.9% was achieved when using SVM as a classifier. Using SVM, the precision recorded was 0.999, with a recall of 0.998, F1-measure of 0.999, and training time of 12,382 s. We can immediately note that the achievement of 99.9% accuracy with a small increase in training time compared to the results reported in Table 4 is justified.

**Figure 8:**
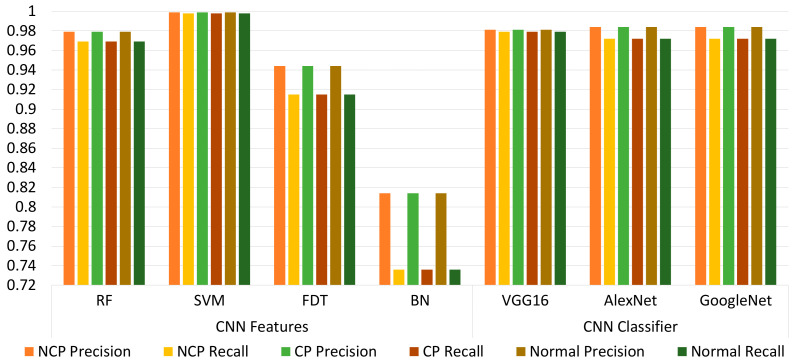
Precision- and recall-based comparison of the proposed method with well-known CNN models.

Figure 8 shows the results of precision and recall comparing using the well-known deep learning algorithms for feature extraction and classification, as well as when using GoogleNet and ResNet18 for feature extraction before inputting the features to the classifiers RF, SVM, FDT, and BN. It can be noted that the highest precision and recall were achieved when using GoogleNet and ResNet18 for feature extraction and when using SVM as a classifier.

**Figure 9:**
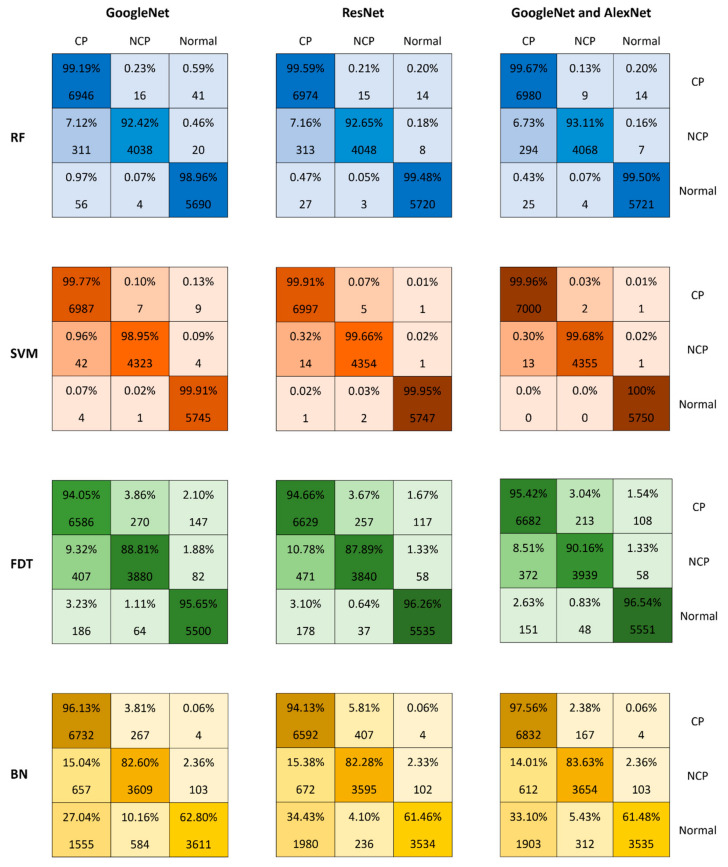
Comparison of the confusion matrix for different classifiers with different deep learning-based feature sets.

Figure 9 shows the confusion matrix when using GoogleNet and ResNet18 for feature extraction and inputting the features to the classifiers RF, SVM, FDT, and BN. The confusion matrix shows both the accuracies achieved for each class and their incorrect predictions. The confusion matrices are also shown when using GoogleNet for feature extraction followed by the classifiers, and when using ResNet18 for feature extraction followed by the classifiers. Comparing all the confusion matrices, we can note that the best accuracies were achieved using the SVM classifier with the 2000 features extracted from both GoogleNet and ResNet18. We can note in this case that the true positive (TP) rates for all the three classes were as follows: NCP with an accuracy of 99.68%, CP with an accuracy of 99.96%, and normal with an accuracy of 100%. The average accuracy for all three classes was 99.9%.

## 7. Discussion

In this paper, a modified DL method was proposed for the accurate automatic detection and diagnosis of COVID-19. This method is not only able to diagnose COVID-19 accurately but also able to differentiate and diagnose COVID-19 from the common flu, including viral pneumonia and bacterial pneumonia, in addition to lung opacity, since they all share similar symptoms. To the authors’ knowledge, the paper is one of the very few targeting the classification of these three classes: novel coronavirus pneumonia (NCP), common pneumonia (CP), and normal. Furthermore, it is one of the few papers combining both CT and X-ray scans of the chest. Therefore, Table 6 shows a comparison between the proposed method and somewhat similar techniques from the extant literature using the same experimental dataset. It is clear that the method proposed in this paper produced superior results to those reported in the extant literature. In the proposed method, more refined hybrid features were used along with the SVM classifier, instead of directly using CNN-based models, which led to better accuracies.

## 8. Conclusions

The need for systems that can automatically detect and diagnose COVID-19 without human intervention is still an urgent priority and will remain so because the same technology can be used for future pandemics and other health conditions. The time-consuming process to monitor and test all individuals for COVID-19 is an impossible task, especially as COVID-19 shares similar symptoms with the common cold and pneumonia. Some off-the-counter tests have been developed and sold, but they are unreliable and add an additional burden because false-positive cases have to visit hospitals and perform specialized diagnostic tests to confirm the diagnosis. The cost analysis of lab tests, time spent on results and diagnosis, and other related costs were not analyzed in this paper, but there is also a cost to these COVID-19 tests, along with the time cost of medical personnel. Automatic machine learning detection and diagnosis can decrease this cost exponentially because only a chest CT scan of the patient is required, with the result reported after a certain computation time. In this paper, we proposed a modified machine learning process that can automatically and accurately detect and diagnose COVID-19. It is among the few studies targeting the classification of three classes: normal, novel coronavirus pneumonia (NCP), and common pneumonia (CP). This is very significant because COVID-19 has symptoms that are similar to the common cold. Combined, this means that the model presented in this paper can accurately detect and diagnose COVID-19, as well as differentiate it from other health issues with similar symptoms. Our proposed modified model achieved the highest average accuracy of 99.9%, exceeding the accuracy reported in the extant literature for similar approaches. Some challenges were encountered during this research, which included finding the right dataset, the distinction between COVID-19 and other medical problems that share similar symptoms, and developing the proposed process through the choice of the right modified DL methods that would extract proper features, the complexity of the system, etc. The end system proposed in this work was able to address all the challenges encountered, and the complete final system was stable with minimal complexity, as indicated by the reduction in training time.

Future work will include increasing the dataset size by combining more open-access datasets and working on feature extraction with machine learning approaches to produce even more accurate results. In addition, future work will include a bibliographical review of COVID-19 detection techniques.

## Figures and Tables

**Table 1:** Summary of the experimental dataset along with sample images.

Class	Total of Patients	Total Images	Sample CT Scan Images		
CP	932	35,191	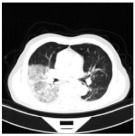	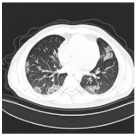	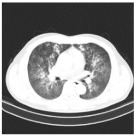
NCP	929	21,872	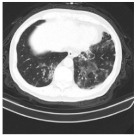	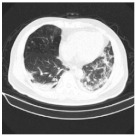	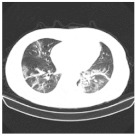
Normal	850	28,548	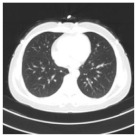	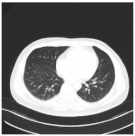	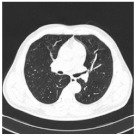

**Table 2:** Experimental results using the most well-known deep learning-based CNN models.

Method	Accuracy	Precision	Recall	F1-Measure	Training Time
AlexNet	98.49	0.986	0.977	0.982	25,568
VGG16	97.32	0.971	0.960	0.972	73,756
GoogleNet	98.71	0.984	0.972	0.978	54,866

**Table 3:** Experimental results using the GoogleNet features with different classifiers.

Method	Accuracy	Precision	Recall	F1-Measure	Training Time
Random forest	93.25	0.932	0.897	0.979	162
Support vector machine	99.61	0.996	0.994	0.997	6027
Fast decision tree	93.25	0.932	0.897	0.979	162
Bayesian network	81.49	0.81	0.729	0.937	178

**Table 4:** Experimental results using the ResNet18 features with different classifiers.

Method	Accuracy	Precision	Recall	F1-Measure	Training Time
Random forest	97.78	0.978	0.967	0.999	202
Support vector machine	99.86	0.999	0.998	0.999	7513
Fast decision tree	93.47	0.999	0.998	0.999	133
Bayesian network	80.14	0.798	0.708	0.93	210

**Table 5:** Experimental results using the combined GoogleNet and ResNet18 features with different classifiers.

Method	Accuracy	Precision	Recall	F1-Measure	Training Time
Random forest	97.93	0.979	0.969	0.999	241
Support vector machine	99.90	0.999	0.998	0.999	12,382
Fast decision tree	94.45	0.944	0.915	0.984	302
Bayesian network	81.88	0.814	0.736	0.931	404

**Table 6:** Comparison of proposed method accuracy with the recent literature using the same dataset.

Reference	Method	Data	Accuracy
Proposed method	Hybrid ResNet18 and GoogleNet 2000 features with SVM	CC-CCII dataset	99.91%
Kang et al. (2020) [49]	A custom-designed 7-layered 3D CNN model	CC-CCII dataset	88.94%
Xing et al. (2020) [50]	Hybrid active learning with 2D U-Net and residual network	CC-CCII dataset	95%
Li et al. (2021) [51]	Hybrid generative adversarial network and DenseNet	CC-CCII dataset	85%
Fu et al. (2021) [52]	Densely connected attention network (DenseNet)	CC-CCII dataset	96.06%
